# Nocturnal Autonomic Dysregulation and Admission-Window Clinical Suicide-Risk Assessment in Hospitalized Children and Adolescents

**DOI:** 10.3390/jcm15176719

**Published:** 2026-08-29

**Authors:** Qiyuan Cao, Jiaqi Xu, Wenjing Li, Yan Zhang, Xuehua Huang, Kexin Zhou, Jinquan Zhang, Lijun Jiang

**Affiliations:** Mental Health Center, West China Hospital, Sichuan University, Chengdu 610041, China; caoqiyuancao@gmail.com (Q.C.); xu_jiaqi008@163.com (J.X.); 15281691971@163.com (W.L.); xuexingyang1988@126.com (Y.Z.); zkx1626@163.com (K.Z.);

**Keywords:** children and adolescents, suicide-risk assessment, heart rate variability, autonomic dysregulation, NGASR, inpatient psychiatry

## Abstract

**Background/Objectives**: Suicide-risk assessment during child and adolescent psychiatric hospitalization draws on patient report, clinical history, and professional observation. Whether nocturnal autonomic physiology is concurrently associated with a structured admission-window assessment after accounting for depressive symptoms and self-reported suicidal ideation remains uncertain. **Methods**: We analyzed 212 hospitalized children and adolescents receiving inpatient care for a major depressive episode. All had a Nurses’ Global Assessment of Suicide Risk (NGASR) rating, self-report measures, covariates, and first admission-night non-contact autonomic data. Principal component analysis was used to derive a nocturnal autonomic dysregulation factor from heart-rate and heart-rate-variability (HRV) summaries. Ordinary least squares regression with HC3 robust standard errors estimated its concurrent association with NGASR after adjustment for age, sex, BMI, Beck Depression Inventory score, and Chinese Beck Scale for Suicide Ideation score. Score-appropriate sensitivity analyses used Poisson, negative-binomial, and predefined NGASR-category models. **Results**: Higher autonomic dysregulation was associated with higher NGASR after adjustment for depressive symptoms and self-reported suicidal ideation (standardized beta = 0.182, 95% CI 0.067 to 0.297; *p* = 0.002; q = 0.005), accounting for an additional 3.2 percentage points of explained variance. The association was similar in a robust Poisson model (incidence-rate ratio = 1.059, 95% CI 1.022 to 1.097; *p* = 0.001) and remained stable after adjustment for subjective sleep quality, AHI, sleep efficiency, and monitoring timing. Separate component models showed larger associations for SDNN and RMSSD than for heart rate or LF/HF, but did not decompose the composite effect. **Conclusions**: Admission-window nocturnal autonomic dysregulation showed a modest concurrent association with clinical suicide-risk assessment after adjustment for depressive symptoms and self-reported suicidal ideation. The finding does not establish disclosure-independent assessment, temporal improvement of admission assessment, or prediction of future suicidal behavior.

## 1. Introduction

Suicide is the second leading cause of death among people aged 10–24 years [1], and the pooled worldwide adolescent suicide mortality rate is approximately 3.77 per 100,000 [2]. These deaths are the most severe outcome of a far more common problem, since suicidal thoughts and behaviors are widespread well before any death. For example, in a national sample of 6483 US adolescents, lifetime prevalence was 12.1% for suicidal ideation, 4.0% for plans, and 4.1% for attempts [3]. This burden makes timely, accurate recognition of elevated risk a clinical priority. Yet recognition is difficult, because the marked heterogeneity of risk profiles undermines reliable risk stratification [4], and contemporary reviews frame adolescent self-harm and suicidality as arising from multiple interacting clinical, developmental, interpersonal, and protective factors rather than from any single observable sign [1,5]. The central question is therefore how to assess such risk.

Self-report is central to suicide-risk assessment [5,6], but measurement choices affect what is counted as suicidality [7], parent and adolescent informants often disagree [8], and some hospitalized adolescents do not disclose suicidal thoughts or behaviors even when asked [9,10]. Stigma and confidentiality concerns can further impede help-seeking [11]. These limitations support a multi-source assessment framework in which patient report remains essential and is interpreted alongside clinical history, professional observation, and other measurements.

In inpatient practice, this complementary information is organized through structured clinical assessment. The Nurses’ Global Assessment of Suicide Risk (NGASR) is a nurse-administered tool that weights recognized suicide-risk indicators to support, rather than replace, clinical judgment [12]. It has been translated and psychometrically evaluated across languages and clinical settings, including acute psychiatric inpatient and emergency services [13,14,15], and a retranslated Chinese version showed acceptable reliability in psychiatric inpatients and correlated with the Beck Scale for Suicide Ideation—Chinese Version [16].

Structured assessment is also imperfect. No single indicator captures suicide risk, and even established predictors show limited individual discrimination [17,18,19,20,21]. Physiological measurements may provide a distinct source of information within multi-source research, but they cannot substitute for patient report or clinical judgment and require evidence that they add reproducible information in a clearly defined assessment window.

The nocturnal period offers a route to such a signal: cardiac autonomic function can be measured unobtrusively during sleep. Reduced heart rate variability (HRV) and diminished parasympathetic tone accompany depression and self-injury in young people. A meta-analysis of children and adolescents with major depression documented altered HRV relative to controls [22], and a meta-analysis of 1979 children and young people linked self-injurious thoughts and behaviors to reduced cardiac autonomic regulation, driven by lower HRV (pooled Hedges’ g = −0.375) [23]. However, this autonomic evidence comes mostly from awake resting-state or stress-task recordings and adult samples [24,25], with the few extended adolescent recordings conducted in ambulatory, out-of-clinic conditions [26]. Reviews note that the relevant indices and time windows remain unresolved and their value for clinical assessment largely untested [27].

To our knowledge, no study has linked first-night nocturnal cardiac autonomic indices to a same-admission-window clinical suicide-risk assessment in hospitalized children and adolescents. The inpatient setting permits low-burden non-contact monitoring during natural sleep. Under-mattress ballistocardiography is an established signal-processing approach [28] that derives cardiac timing information from body motion without requiring active participation during acquisition. In this study, however, the admission-day NGASR assessment preceded the first-night recording; the design therefore evaluates a concurrent admission-window association rather than whether physiology improved an assessment that had already been completed.

To address this gap, we examined whether greater nocturnal autonomic dysregulation was associated with the concurrent NGASR score in 212 hospitalized children and adolescents aged 9–18 years after adjustment for depressive symptoms, self-reported suicidal ideation, age, sex, and BMI. The exposure was a first-night nocturnal autonomic factor recorded with a non-contact device. We hypothesized a positive adjusted association with NGASR, without prespecifying a clinical cutoff or a prospective prediction claim.

## 2. Materials and Methods

### 2.1. Study Design and Setting

We conducted a cross-sectional observational study of hospitalized children and adolescents receiving inpatient psychiatric care for a major depressive episode. On admission day 1, trained psychiatric nurses completed the NGASR and participants completed baseline self-report measures; nocturnal autonomic function was then recorded during the first available admission-night monitoring session. Because the physiological recording followed the admission-day assessment, the analysis tested a concurrent admission-window association and did not evaluate whether physiology improved the already completed admission assessment.

The study was conducted at the Mental Health Center of West China Hospital, Sichuan University. In the analytic sample, index admissions occurred between May 2025 and February 2026. The analytic design used the first admission-night non-contact recording, the NGASR rating obtained on admission day 1, and self-report scales completed on the first day of admission. Reporting follows STROBE principles for observational studies and RECORD-style transparency for clinically collected and device-derived data [29,30].

### 2.2. Participants

Eligible participants were children and adolescents aged 9 to 18 years admitted for inpatient psychiatric care at the Mental Health Center, West China Hospital, during the enrollment window (May 2025 to February 2026).

Inclusion criteria were: (1) a psychiatrist-assigned major depressive episode diagnosed according to DSM-5 criteria; (2) clinical stability sufficient to complete overnight monitoring, self-report questionnaires, and the clinician and nursing assessments; (3) adequate comprehension and cooperation to complete the study assessments; and (4) written informed consent from a legal guardian and assent/consent from the participant.

Exclusion criteria were: (1) current severe psychotic symptoms, severe manic excitement, or aggression; (2) serious neurological or other physical illness, or any other condition likely to affect nocturnal physiology or mood assessment; (3) an uncontrolled primary sleep disorder likely to confound monitoring; (4) frequent recent changes in psychiatric or sleep-affecting medication that could destabilize assessment; (5) substance use disorder or recent psychoactive substance use; (6) moderate-to-severe intellectual disability or severe language or cognitive impairment precluding questionnaire completion or cooperation; (7) inability or unwillingness to complete the overnight monitoring; and (8) other circumstances judged by the investigators to make participation unsuitable.

All participants and their guardians provided assent and written informed consent, as described in the Section 2.3.

**Figure 1:**
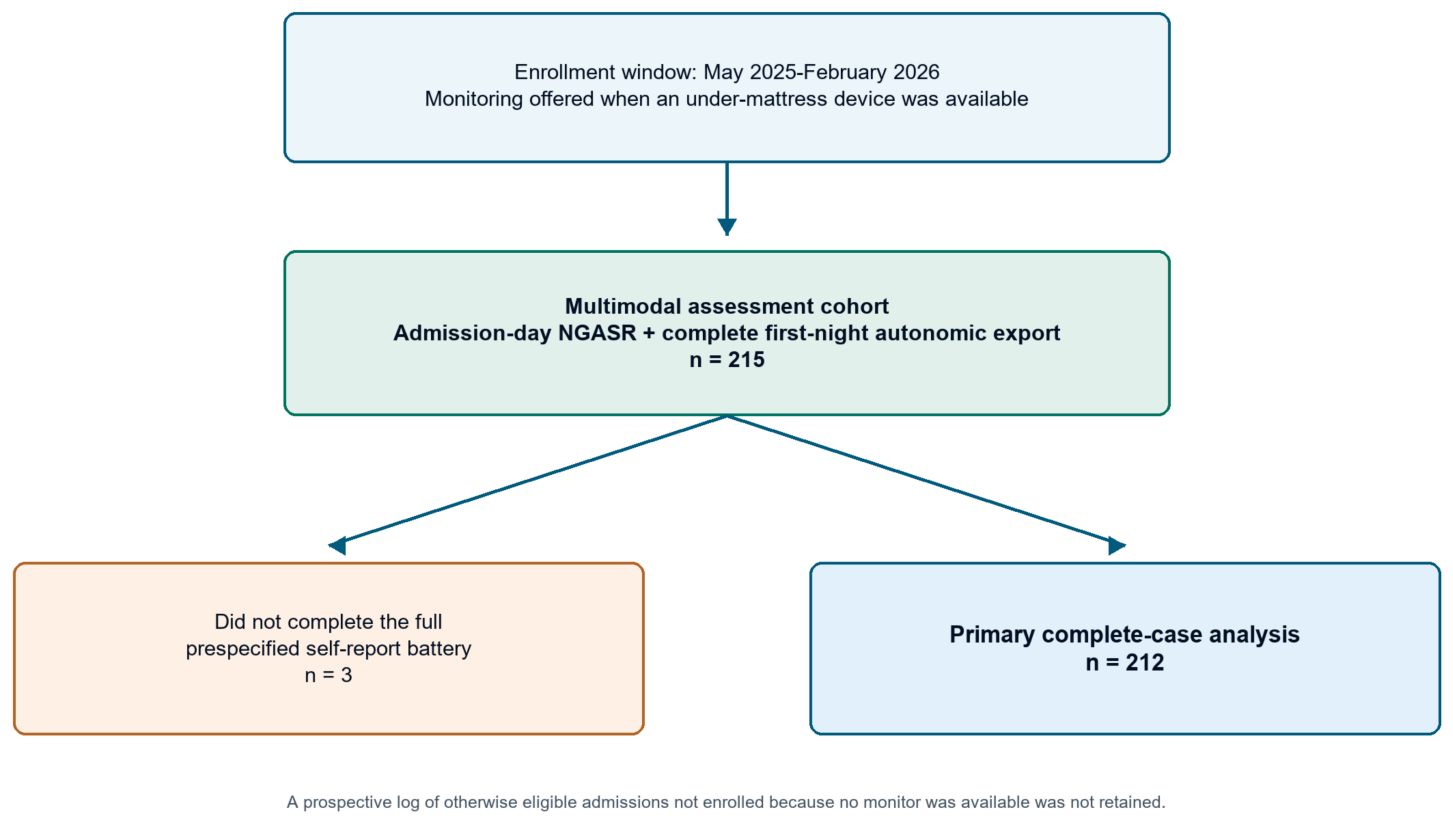
Study participant flow. During the May 2025 to February 2026 enrollment window, 215 children and adolescents entered the multimodal assessment cohort with an admission-day NGASR rating and a complete first-night export for all four autonomic indicators. Three did not complete the full prespecified self-report battery, leaving 212 participants in the primary complete-case analysis. No additional participant was excluded for device-quality reasons after cohort entry. A prospective log of otherwise eligible admissions not enrolled when a monitor was unavailable was not retained.

During the enrollment window (May 2025 to February 2026), admission-night monitoring was offered when one of a limited number of under-mattress monitors was available. A total of 215 children and adolescents entered the multimodal assessment cohort; all had an admission-day NGASR rating and a complete first-night export for the four autonomic indicators. Three participants did not complete the full prespecified self-report battery and were excluded from the complete-case model, leaving 212 participants for analysis (Figure 1). Because a prospective screening log of otherwise eligible ward admissions not enrolled when a monitor was unavailable was not retained, that upstream count could not be reconstructed; availability-based recruitment is therefore acknowledged as a potential source of selection bias.

### 2.3. Ethics

The study was conducted in accordance with the Declaration of Helsinki and approved by the Biomedical Ethics Review Committee of West China Hospital, Sichuan University (approval no. 2024 Ethics Review No. 1937; approval date: 22 October 2024). Written informed consent was obtained from legal guardians, and adolescent participants provided written assent/consent before participation. Participant-facing materials describe voluntary participation, confidentiality, the right not to complete assessments or to withdraw without affecting care, and authorization for clinical and research/statistical use.

### 2.4. Outcome: Clinical and Nursing Suicide-Risk Assessment

The primary outcome was the Nurses’ Global Assessment of Suicide Risk (NGASR), completed by nursing staff on admission day 1. The original NGASR is a 15-item weighted clinical assessment index designed to structure suicide-risk assessment and support, rather than replace, clinical judgment [12]. Conventionally reported strata are low risk (5 or lower), intermediate risk (6–8), high risk (9–11), and very high risk (12 or higher) [13,14]. The center used the standard Chinese-language 15-item NGASR [16]. Its indicators include hopelessness, expressed suicidal intent or plan, prior attempts, and recent loss; it contains no physiological item. Although completed by nurses, several indicators rely on patient statements and documented clinical history. NGASR was therefore treated as a structured admission-window clinical assessment score, not as disclosure-independent information or a future suicidal-behavior endpoint.

### 2.5. Self-Report Measures and Clinical Anchor

Depressive symptom severity was measured using the Chinese version of the Beck Depression Inventory (BDI) total score. Patient-reported suicidal ideation was assessed using the Chinese version of the Beck Scale for Suicide Ideation (BSI-CV), which is based on the original Scale for Suicide Ideation and Chinese-version validation work [31,32]. The BSI-CV contains 19 items rated from 0 to 2. The first five screening items assess suicidal ideation and yield a score ranging from 0 to 10, with higher scores indicating more severe suicidal ideation. In the present analysis, BSI-CV suicidal ideation was operationalized as the sum of the five suicidal-ideation screening items according to the questionnaire scoring rule. Baseline self-report measures were completed on the first day of admission.

The Columbia-Suicide Severity Rating Scale (C-SSRS), which has multisite validation evidence across adolescents and adults [33], was available in the database as a baseline three-level clinical risk classification (low, moderate, or high). It was used only as an alternative clinical anchor. For comparability with the primary analysis, the recorded 1–3 score was analyzed with OLS-HC3; an ordinal logistic model was added as a score-appropriate sensitivity analysis.

### 2.6. Nocturnal Autonomic Measures

Nocturnal physiological data were collected during the first admission night with an L-800 non-contact under-mattress monitor provided by Chengdu Langzhi Digital Medical and Nursing Care Technology Co., Ltd. (Chengdu, China). Its polyvinylidene fluoride piezoelectric sensor acquired a ballistocardiographic signal from which the proprietary processing pipeline exported nightly summary heart rate (beats/min), SDNN (ms), RMSSD (ms), and LF/HF. The analytic dataset contained these summaries but not raw BCG or RR/NN interval series, and the software/algorithm version was unavailable in the retained study records. The summaries were therefore treated as device-derived autonomic indicators whose measurement properties depend on the specific signal-processing pipeline [34,35].

The analysis used one admission-night export per participant and did not aggregate across nights. Device-provider documentation described a qualified overnight export as total recording duration ≥ 4 h, valid-signal coverage ≥ 70%, longest continuous valid nocturnal segment ≥ 3 h, out-of-bed time ≤ 20% of recording duration, and fewer than 1000 device-flagged snoring-related artifacts. Body-movement and out-of-bed periods were distinguished from valid signal by BCG amplitude and were excluded by the proprietary processing pipeline. Segment-level flags were unavailable for independent reprocessing. All 215 participants at cohort entry had the four required exported autonomic summaries; the analysis team applied no additional device-quality exclusion after cohort entry.

Published studies of other under-mattress and non-contact systems demonstrate the feasibility of deriving cardiac and sleep-related measures from ballistocardiographic signals [36,37,38,39], and ECG/PPG studies inform general HRV measurement considerations [40]. These studies do not validate the L-800 device or its proprietary pipeline. Accordingly, the present outputs are described as device-derived nocturnal summaries rather than polysomnographic endpoints or ECG-equivalent HRV measurements.

The primary exposure was a nocturnal autonomic dysregulation factor derived from the four prespecified exported summaries. The indicators were winsorized at the 1st and 99th percentiles and z-standardized before principal component analysis within the 212-person analytic sample. No magnitude threshold was used to retain or exclude indicators. Pairwise correlations were 0.949 for SDNN with RMSSD, −0.696 for SDNN with heart rate, −0.368 for SDNN with LF/HF, −0.709 for RMSSD with heart rate, −0.504 for RMSSD with LF/HF, and 0.495 for heart rate with LF/HF. PC1 had an eigenvalue of 2.901 and explained 72.5% of total variance; the remaining eigenvalues were 0.708, 0.352, and 0.038. Oriented PC1 component weights (eigenvector coefficients) were −0.537 for SDNN, −0.557 for RMSSD, 0.504 for nocturnal heart rate, and 0.383 for LF/HF. Their squared values summed to 1.000, and these coefficients were used directly to compute the PC1 score. Higher scores therefore represented lower time-domain HRV and higher nocturnal heart rate. LF/HF was retained as the prespecified frequency-domain complement but interpreted cautiously because of its smaller absolute component weight and dependence on measurement and sleep state.

### 2.7. Covariates

All primary models adjusted for age, sex, BMI, BDI total score, and BSI-CV suicidal ideation. The available medication fields contained discharge medication classes and could not reliably characterize psychotropic exposure before admission or before the first-night recording; medication was therefore not included as a covariate, and residual confounding by prior psychotropic treatment is acknowledged. Additional sensitivity models separately added PSQI total, device-derived AHI, sleep efficiency, and monitoring timing, followed by combined sleep-context specifications. Sleep staging and independently defined sleep duration were unavailable and were not imputed.

### 2.8. Statistical Analysis

Participant characteristics are summarized as means and standard deviations for continuous variables and counts and percentages for categorical variables. The primary association between nocturnal autonomic dysregulation and continuous NGASR was estimated using ordinary least squares regression. Residual distribution, heteroscedasticity, functional form, influence, and variance-inflation diagnostics were examined; HC3 robust standard errors were used for primary inference.

Four nested models were used to show how the association changed after demographic and patient-reported measures were accounted for: Model 0 included the autonomic factor; Model 1 added age, sex, and BMI; Model 2 added BDI; and Model 3 added BSI-CV suicidal ideation. Model 3 estimated the concurrent association with NGASR after adjustment for these prespecified variables; it was not interpreted as establishing independence from disclosure.

The primary estimate was the standardized beta coefficient for the autonomic dysregulation factor in Model 3, reported with a 95% confidence interval (CI), *p* value, and false-discovery-rate-adjusted q value. Confidence intervals and *p* values were calculated using HC3 robust standard errors. The q values were computed with the Benjamini–Hochberg false-discovery-rate (BH-FDR) procedure, applied within a single exposure-and-component family: the autonomic dysregulation factor and its four components (nocturnal heart rate, SDNN, RMSSD, and LF/HF). For clinical interpretability, we also report the estimated difference in NGASR points per 1-SD increase in autonomic dysregulation. Incremental model fit was summarized using change in R-squared (Delta R2) comparing each exposure-added model with the self-report model containing age, sex, BMI, BDI, and BSI-CV suicidal ideation.

Component analyses examined nocturnal heart rate, SDNN, RMSSD, and LF/HF separately under the final adjustment set. These models were used to describe direction and relative effect-size magnitude, not to decompose the composite effect or estimate each component’s contribution. Score-appropriate sensitivities fitted robust Poisson and negative-binomial count models and a proportional-odds model using the four predefined NGASR strata. Proportional-odds plausibility was assessed descriptively by comparing the autonomic-factor coefficient across the three cumulative binary-logit models; this comparison was not a formal Brant test. Because the cutoff-specific coefficients varied materially, the common odds ratio was treated as a bounded sensitivity and interpreted cautiously rather than as evidence of a uniform shift across risk strata. The primary continuous NGASR-score OLS analysis did not rely on the proportional-odds assumption. Additional models adjusted for PSQI, AHI, sleep efficiency, monitoring timing, and combined sleep context. The three-level C-SSRS risk classification was analyzed as an alternative clinical anchor.

## 3. Results

### 3.1. Participant Characteristics

This study included 212 hospitalized children and adolescents. The mean age was 14.9 years (SD 1.7), and 173 participants (81.6%) were female. Mean BMI was 20.6 (SD 4.6). Mean BDI was 33.4 (SD 15.0), mean BSI-CV suicidal ideation was 6.0 (SD 3.1), and mean NGASR was 9.0 (SD 2.8). NGASR strata included 30 low-, 47 intermediate-, 97 high-, and 38 very-high-risk assessments; C-SSRS categories included 40 low-, 55 moderate-, and 117 high-risk assessments. All 212 met DSM-5 criteria for a major depressive episode at admission. Baseline characteristics are summarized in Table 1. Unadjusted contrasts across conventional NGASR strata are shown in Supplementary Figure S1.

### 3.2. Main Association Between Nocturnal Autonomic Dysregulation and NGASR

Higher nocturnal autonomic dysregulation was associated with higher NGASR scores across the nested model sequence. In the unadjusted model, each 1-SD higher autonomic dysregulation factor corresponded to an estimated 0.496-point higher NGASR score. The association remained similar after adjustment for age, sex, and BMI, and after further adjustment for BDI total score.

**Figure 2:**
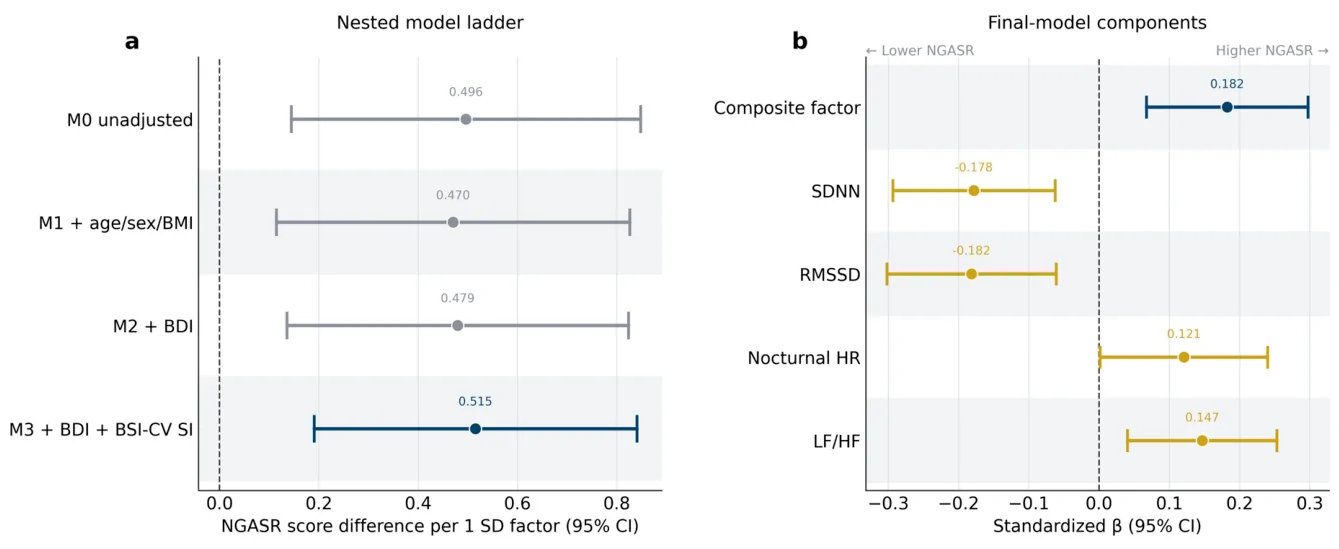
Incremental association between first admission-night nocturnal autonomic dysregulation and NGASR (*n* = 212). (**a**) Nested model ladder showing the NGASR-point difference per 1-SD higher autonomic factor from the unadjusted model through Model 3 (age, sex, BMI, BDI, and BSI-CV suicidal ideation). (**b**) Separate adjusted standardized estimates for the composite factor and its four components. The component models describe direction and effect-size magnitude but do not partition the composite association or establish relative contribution. Points to the right of zero indicate higher NGASR. Abbreviations: NGASR, Nurses’ Global Assessment of Suicide Risk; BDI, Beck Depression Inventory; BSI-CV, Beck Scale for Suicide Ideation, Chinese version; SDNN, RMSSD, and LF/HF, heart-rate-variability indices.

In the primary adjusted model, which included age, sex, BMI, BDI, and BSI-CV suicidal ideation, the standardized beta coefficient for the autonomic factor was 0.182 (95% CI, 0.067 to 0.297; *p* = 0.002; q = 0.005). In NGASR-point units, each 1-SD higher factor corresponded to an estimated 0.515-point higher NGASR score (95% CI, 0.190 to 0.840). Adding the factor increased explained variance by 3.2 percentage points (change in R-squared, 0.032) relative to the covariate-only model. Detailed nested and component estimates are reported in Table 2 and visualized in Figure 2.

### 3.3. Component Analyses

The four autonomic summaries were examined separately under the same adjustment set. Lower SDNN (standardized beta = −0.178; 95% CI, −0.294 to −0.063; *p* = 0.003), lower RMSSD (beta = −0.182; 95% CI, −0.302 to −0.061; *p* = 0.003), higher LF/HF (beta = 0.147; 95% CI, 0.040 to 0.253; *p* = 0.007), and higher nocturnal heart rate (beta = 0.121; 95% CI, 0.001 to 0.240; *p* = 0.047) were directionally consistent with the composite. SDNN and RMSSD had larger separate adjusted effect estimates than nocturnal heart rate, but these regressions did not decompose the composite effect and do not support a claim that any component accounted for a defined proportion of it.

### 3.4. Sensitivity Analyses

Sensitivity analyses supported the primary concurrent association. A robust Poisson model yielded an incidence-rate ratio of 1.059 per 1-SD higher autonomic factor (95% CI, 1.022 to 1.097; *p* = 0.001); the negative-binomial result was nearly identical because estimated overdispersion was negligible. The four-stratum proportional-odds model yielded a common odds ratio of 1.392 (95% CI, 1.070 to 1.811; *p* = 0.014). However, the cumulative-cutpoint coefficients were 0.541, 0.448, and 0.061 for the intermediate-or-higher, high-or-higher, and very-high-risk cutoffs, respectively; at the very-high-risk cutoff, the odds ratio was 1.063 (95% CI, 0.735 to 1.539; *p* = 0.744). This nonuniformity made the proportional-odds assumption questionable, so the common odds ratio should be interpreted cautiously and not as evidence of a uniform category shift. The primary continuous-score OLS analysis did not rely on this assumption. Across models adding PSQI, AHI, sleep efficiency, monitoring timing, and combined sleep context, standardized beta estimates ranged from 0.182 to 0.198. These sleep-context sensitivity estimates are summarized in Supplementary Figure S2 and Supplementary Table S1. For the C-SSRS alternative anchor (40 low-, 55 moderate-, and 117 high-risk ratings), the OLS-HC3 standardized beta was 0.145 (95% CI, 0.023 to 0.267; *p* = 0.020) and the ordinal-logistic odds ratio was 1.429 (95% CI, 1.068 to 1.913; *p* = 0.016).

For the primary Model 3, residuals showed mild non-normality (Jarque–Bera *p* = 0.008), supporting HC3 inference. The Breusch–Pagan test (*p* = 0.455) and Ramsey RESET test (*p* = 0.387) did not indicate major heteroscedasticity or functional-form misspecification; the maximum variance-inflation factor was 2.14 and the maximum Cook’s distance was 0.076.

## 4. Discussion

### 4.1. Principal Finding and Its Components

First-night nocturnal autonomic dysregulation showed a modest concurrent association with admission-window NGASR after adjustment for age, sex, BMI, depression severity, and self-reported suicidal ideation (standardized beta 0.182; 95% CI 0.067 to 0.297; *p* = 0.002; q = 0.005), adding about 3.2 percentage points of explained variance. Because several NGASR indicators draw on patient statements and documented history, this result does not establish a disclosure-independent assessment channel. Separate component models showed larger adjusted effect estimates for SDNN and RMSSD than for nocturnal heart rate, but they did not partition the composite effect. The result should also be interpreted against a developing and sex-patterned autonomic baseline [41,42]; the predominantly female, single-center cohort limits inference about male patients and other settings.

### 4.2. Interpretive Context and Measurement Limits

Lower whole-night SDNN and RMSSD may be compatible with reduced parasympathetic modulation or greater autonomic arousal, but the present data cannot identify a stage-specific mechanism. Whole-night summaries combine wakefulness and multiple sleep stages, and the BCG-derived pipeline was not validated against ECG or PSG in this sample. Prior sleep-HRV and adolescent-depression studies provide biological context [43,44,45,46], but specific recovery or downregulation mechanisms remain hypotheses for future stage-resolved studies rather than conclusions of this analysis. Inflammatory pathways were not measured and are not inferred here.

### 4.3. A Measurement That Fits the Admission Window

The practical advantage of the exposure is low-burden acquisition during inpatient sleep. That advantage does not change the temporal order: the admission-day NGASR was completed before the first-night recording, so the study cannot show that physiology improved the assessment already performed. It instead shows that a device-derived autonomic profile measured during the same admission window was associated with the structured clinical score. Future longitudinal work could test whether repeated recordings predict subsequent clinically meaningful outcomes or improve decisions when incorporated prospectively.

Non-contact monitoring may facilitate repeated-measures research when laboratory polysomnography is impractical [47,48]. Translation would nevertheless require validation of the actual device and processing pipeline, prespecified quality-control rules, repeated-night reliability, and prospective evaluation against clinically meaningful outcomes. The present association alone does not establish real-time alerting, screening performance, or incremental clinical utility.

### 4.4. Clinical Interpretation

The physiological recording itself did not require active patient reporting during the night, but the NGASR outcome incorporated patient statements, clinical history, and nursing judgment. The result is therefore best understood as a modest concurrent association between two differently acquired admission-window information sources, not as evidence that physiology can reveal undisclosed risk. Any future clinical application would require prospective prediction, calibrated decision thresholds, and demonstrated benefit beyond standard assessment.

### 4.5. Limitations

Several caveats bound these findings. The design is cross-sectional and same-window, precluding temporal, causal, or prospective-risk inference; the NGASR is a structured clinical assessment rather than a suicidal-behavior endpoint. The admission-day rating preceded the first-night recording, so the study did not test whether physiology improved admission assessment. Enrollment depended on availability of a limited number of under-mattress monitors, and a prospective log of otherwise eligible admissions not enrolled when no monitor was available was not retained; upstream coverage could not be quantified, and availability-based recruitment may have introduced selection bias. Three of 215 cohort participants did not complete the full self-report battery, leaving 212 complete cases. Only discharge medication-class fields were available, so psychotropic exposure before admission or before the first-night recording could not be characterized reliably. The single-night, whole-night summaries did not resolve sleep stages or night-to-night reliability. Raw BCG or RR/NN data and the software/algorithm version were unavailable, and the L-800 export was not validated against ECG or PSG in this sample. The single-center, predominantly female cohort limits generalizability.

## 5. Conclusions

In hospitalized children and adolescents, a first-night device-derived autonomic profile showed a modest concurrent association with admission-window NGASR after adjustment for depressive symptoms and self-reported suicidal ideation. The finding does not establish disclosure independence, temporal improvement of assessment, or prediction of future suicidal behavior. Replication should use transparent device documentation, stage-aware or ECG-referenced measurement, repeated recordings, and prospective clinical outcomes.

## Figures and Tables

**Table 1:** Baseline characteristics of the analytic sample.

Characteristic	Overall Sample (*n* = 212)
Age, years	14.9 (1.7); range 9–18
Female sex, *n* (%)	173 (81.6)
BMI, kg/m^2^	20.6 (4.6)
Major depressive episode, *n* (%)	212 (100)
BDI total score	33.4 (15.0)
BSI-CV suicidal ideation	6.0 (3.1)
NGASR total score	9.0 (2.8)
Nocturnal heart rate, bpm	69.7 (10.2)
Nocturnal SDNN, ms	43.9 (15.5)
Nocturnal RMSSD, ms	51.0 (23.1)
Nocturnal LF/HF	0.6 (0.6)
NGASR strata, low/intermediate/high/very high, *n*	30/47/97/38
C-SSRS risk category, low/moderate/high, *n*	40/55/117

Notes: Continuous variables are summarized as mean (SD), and categorical variables as *n* (%). Abbreviations: BMI, body mass index; BDI, Beck Depression Inventory; BSI-CV, Beck Scale for Suicide Ideation, Chinese version; NGASR, Nurses’ Global Assessment of Suicide Risk; SDNN/RMSSD/LF-HF, heart-rate-variability indices.

**Table 2:** **Multivariable associations between nocturnal autonomic dysregulation and NGASR.** Panel A. Nested model sequence for the autonomic dysregulation factor. Panel B. Primary exposure and autonomic component estimates in the final adjustment set.

**(A)**							
**Model**	**Adjustment**	***n***	**Standardized β (95% CI)**	**Unstandardized B (95% CI)**		***p***	**R2**
M0	Autonomic factor only	212	0.175 (0.051 to 0.300)	0.496 (0.145 to 0.848)		0.006	0.031
M1	+age/sex/BMI	212	0.166 (0.040 to 0.292)	0.470 (0.114 to 0.826)		0.010	0.036
M2	+BDI	212	0.170 (0.048 to 0.291)	0.479 (0.135 to 0.824)		0.006	0.122
M3	+BDI + BSI-CV	212	0.182 (0.067 to 0.297)	0.515 (0.190 to 0.840)		0.002	0.190
**(B) Separate adjusted component models**							
**Exposure**		**Standardized β (95% CI)**		**Unstandardized B (95% CI)**	***p***	**BH-FDR q**	**Delta R2**
Autonomic dysregulation factor		0.182 (0.067 to 0.297)		0.515 (0.190 to 0.840)	0.002	0.005	0.032
SDNN		−0.178 (−0.294 to −0.063)		−0.504 (−0.831 to −0.177)	0.003	0.005	0.031
RMSSD		−0.182 (−0.302 to −0.061)		−0.514 (−0.854 to −0.173)	0.003	0.005	0.032
Nocturnal heart rate		0.121 (0.001 to 0.240)		0.341 (0.004 to 0.678)	0.047	0.047	0.013
LF/HF		0.147 (0.040 to 0.253)		0.415 (0.114 to 0.715)	0.007	0.009	0.020

Notes: The outcome is NGASR. Model 3 is adjusted for age, sex, BMI, BDI total score, and BSI-CV suicidal ideation. The autonomic dysregulation factor was refitted within the analytic sample. Unstandardized B values represent differences in NGASR points per 1-SD higher exposure. Component rows are included to show the direction of the composite exposure and should be interpreted as secondary explanatory estimates. *p* values use HC3 robust standard errors; q values are BH-FDR adjusted within the exposure/component family. Delta R2 compares each exposure-added model with the self-report model containing age, sex, BMI, BDI, and BSI-CV suicidal ideation (R-squared 0.158). In Panel A, SI denotes suicidal ideation.

## Data Availability

The data presented in this study are available on request from the corresponding author. The data are not publicly available due to privacy and ethical restrictions.

## Supplementary Materials

The following supporting information can be downloaded at https://www.mdpi.com/article/10.3390/jcm15176719/s1: Figure S1: NGASR group contrasts in nocturnal physiology; Figure S2: sensitivity analyses; Table S1: Sleep-context sensitivity analyses.
