# Supplementary material for: Nocturnal Autonomic Dysregulation and Admission-Window Clinical Suicide-Risk Assessment in Hospitalized Children and Adolescents
Source: J Clin Med. 2026 Aug 29;15(17):6719. doi: 10.3390/jcm15176719 (PMC13566821; doi:10.3390/jcm15176719)
Supplement: Supplementary file 1 [file jcm-15-06719-s001.zip › jcm-4452044-supplementary.pdf]

Supplementary Materials

Nocturnal Autonomic Dysregulation and Admission-Window Clinical Suicide-Risk Assessment in Hospitalized Children and Adolescents

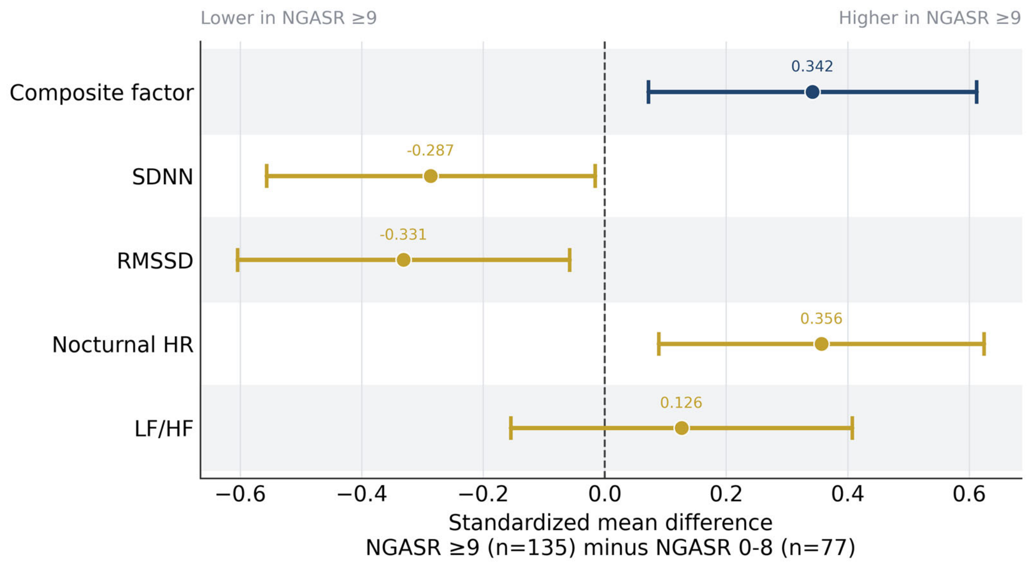

Supplementary Figure S1. NGASR group contrasts in nocturnal physiology. Nocturnal autonomic differences between higher- and lower-risk groups defined by the NGASR (n=212). Standardized mean differences (95% CI) compare children and adolescents with NGASR >=9 (higher clinically assessed risk; n=135) with those scoring 0-8 (n=77), for the composite autonomic dysregulation factor and its four components. The higher-risk group shows a higher composite factor and nocturnal heart rate and lower SDNN and RMSSD, consistent with the primary regression, whereas LF/HF showed a weaker, non-significant group difference. The cut at 9 corresponds to the conventional NGASR high-risk stratum boundary. Points to the right of zero indicate higher values in the NGASR >=9 group.

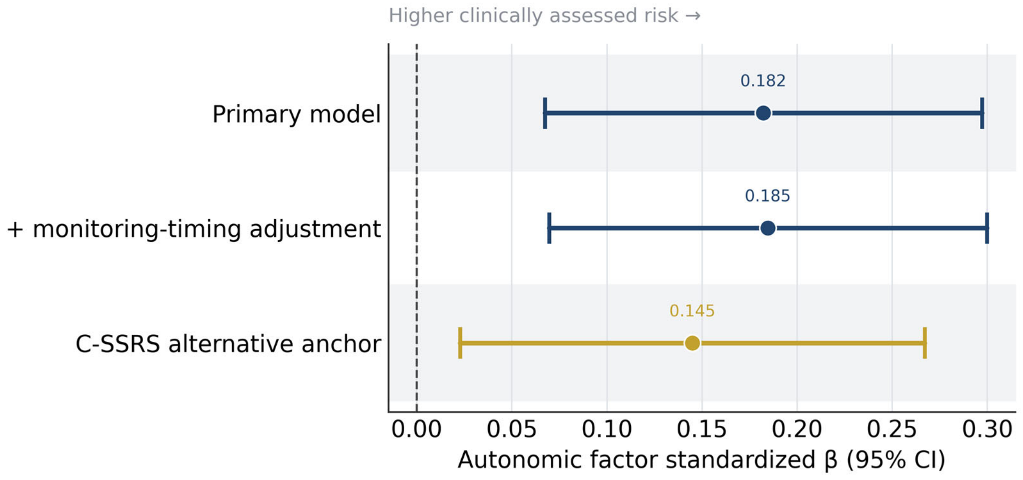

Supplementary Figure S2. Sensitivity analyses. Sensitivity analyses for the association between first admission-night nocturnal autonomic dysregulation and suicide-risk assessment (n=212). Standardized beta (95% CI) for the autonomic dysregulation factor are shown for the primary model (0.182), the same model with additional adjustment for monitoring timing (0.185), and a model using the C-SSRS clinical risk rating as an alternative outcome anchor (0.145). The estimate is stable across specifications and remains above zero. The adjustment set was age, sex, BMI, BDI, and BSI-CV suicidal ideation.

Supplementary Table S1. Sleep-context sensitivity analyses.

Model	Added variable(s)	n	Standardized β	95% CI	p
Primary	None beyond the primary adjustment set	212	0.182	0.067 to 0.297	0.002
Subjective sleep quality	PSQI total score	212	0.195	0.081 to 0.309	0.001
Sleep-disordered breathing	Device-derived AHI	212	0.182	0.068 to 0.297	0.002
Sleep continuity	Device-derived sleep efficiency	212	0.183	0.068 to 0.297	0.002
Monitoring timing	Days from admission to monitoring	212	0.185	0.070 to 0.300	0.002
Combined objective sleep context	AHI, sleep efficiency, and monitoring timing	212	0.186	0.072 to 0.301	0.001
Combined subjective and objective sleep context	PSQI, AHI, sleep efficiency, and monitoring timing	212	0.198	0.083 to 0.313	0.001

Note: All models used ordinary least squares with HC3 robust standard errors and included age, sex, BMI, BDI total score, and BSI-CV suicidal ideation. PSQI, Pittsburgh Sleep Quality Index; AHI, apnea-hypopnea index.
